# An Overview of the Reimbursement Decision-Making Processes in Bulgaria As a Reference Country for the Middle-Income European Countries

**DOI:** 10.3389/fpubh.2018.00061

**Published:** 2018-03-05

**Authors:** Maria Kamusheva, Mariya Vassileva, Alexandra Savova, Manoela Manova, Guenka Petrova

**Affiliations:** ^1^Faculty of Pharmacy, Medical University of Sofia, Sofia, Bulgaria; ^2^National Council on Prices and Reimbursement, Sofia, Bulgaria

**Keywords:** reimbursement, Bulgaria, low and middle-income Balkan countries, innovative medicines, access, affordability, positive drug list

## Abstract

**Background:**

Policy makers face a lot of challenges in the process of drug reimbursement decision-making, especially in the context of entering the market of more and more innovative medicinal products (MPs). The aim of the current study is to make an overview of the reimbursement system development and to evaluate the access of innovative medicines, which have entered the EU-market in the period 2015–2017, in Bulgaria as reference example for middle-income European country.

**Methods:**

A literature and a legislative systematic review regarding the Bulgarian reimbursement system as well as a defining the number of available innovative reimbursed MPs in 2017 in Bulgaria was made.

**Results:**

The reimbursement legislation in Bulgaria is quite unstable due to constant changes, which have been made, especially in the recent years. Despite this fact, the reimbursement process in Bulgaria is in accordance with the Transparency Directive. Bulgarian patients have a relatively delayed access to innovative medicines as only 5% of centrally authorized MPs in 2017 are available in the positive drug list (PDL), 16% of all in 2016 and 18%—in 2015. This could be explained by the long procedure for their appraisal in Bulgaria: the first step is issuing an opinion by the HTA Committee, followed by negotiation of discounts between the marketing authorization holder and the National Health Insurance Fund and making a final decision by the National Council on Prices and Reimbursement (NCPR) for the inclusion into the PDL.

**Conclusion:**

Optimization of the procedure for issuing reimbursement status for innovative MPs is needed, such as improvements in the process of conducting HTA reports and their appraisal, incorporation of adequate systems for following the effectiveness and safety of MPs in the real-world conditions, value-based pricing implementation, and increasing the financial control over the health insurance system.

## Introduction

The policy makers are constantly facing the challenge to find the balance between the increased patients’ needs of innovative, high costly medicines and limited financial resources (1). The scarce resources and the increasing patients’ needs define the need for implementation of strict pharmacoeconomic evaluations for the purposes of making the right decision.

A lot of issues still exist, notably in the middle and upper-middle-income European countries (2). The economic situation in these countries is critical and there is an emergency need of more efficient reallocation of the resources especially in the pharmaceutical sector. Their health-care systems are not as stable as they should be due to a lot of reforms which have been made in the recent years (2). Rancic et al. concluded that the total health expenditures showed significant growth in the period 1995–2012 probably due to population aging (3). Pharmaceutical expenditures are a significant part of total health-care expenditures. For example, in Bulgaria the pharmaceutical expenditures increase every year, which leads to the annual budget deficit for National Health Insurance Fund (NHIF) (4). Therefore, more precise cost-containment measures should be applied as well as optimization of HTA usage in order to get better value for money (2, 5). Implementation of effective working generic policy and entering the market of biosimilar products are also possible measures (2). As Jakovljevic et al. highlighted there are some factors such as demographic crisis which could not be overcome and which is a main pharmaceutical expenditures driver in the next years (2, 6–8). Moreover, Bulgaria as the EU Member State with the lowest income per capita [only 47% of the EU average (9)] faces many challenges in ensuring the most innovative medicines for its citizens.

The aim of the current study is to make an overview of the reimbursement system development and to evaluate the access of innovative medicines, which have entered the EU-market in the period 2015–2017, in Bulgaria as reference example for middle-income European country.

## Materials and Methods

The first part of the study was a literature and a legislative systematic review regarding the implemented reimbursement system in Bulgaria for the period 2000–2017. A search was made in the official websites of Bulgarian institutions such as Ministry of Health, NHIF, National Council on Prices and Reimbursement of Medicinal Products (MPs), National Centre for Public Health and Analyses, and Bulgarian Drug Agency in order to identify the latest legislative documents and guidelines for conducting of administrative pricing and reimbursement procedures.

The second part of the study included a search of all MPs (MPs) which received marketing authorization through the centralized procedure for the period 2015–2017. A comparison of the generated list of these MPs by the website of the European Medicines Agency (EMA) and the current Bulgarian Positive Drug List (PDL) was made. Therefore, the availability of the newest medicines in Bulgaria was analyzed.

The third part of the study presents a systematic and analytical review of the identified issues in the reimbursement process in Bulgaria on the basis of the authors’ point of view and officially published scientific studies.

## Results

### Reimbursement Legislation in Bulgaria

**Figure 1:**
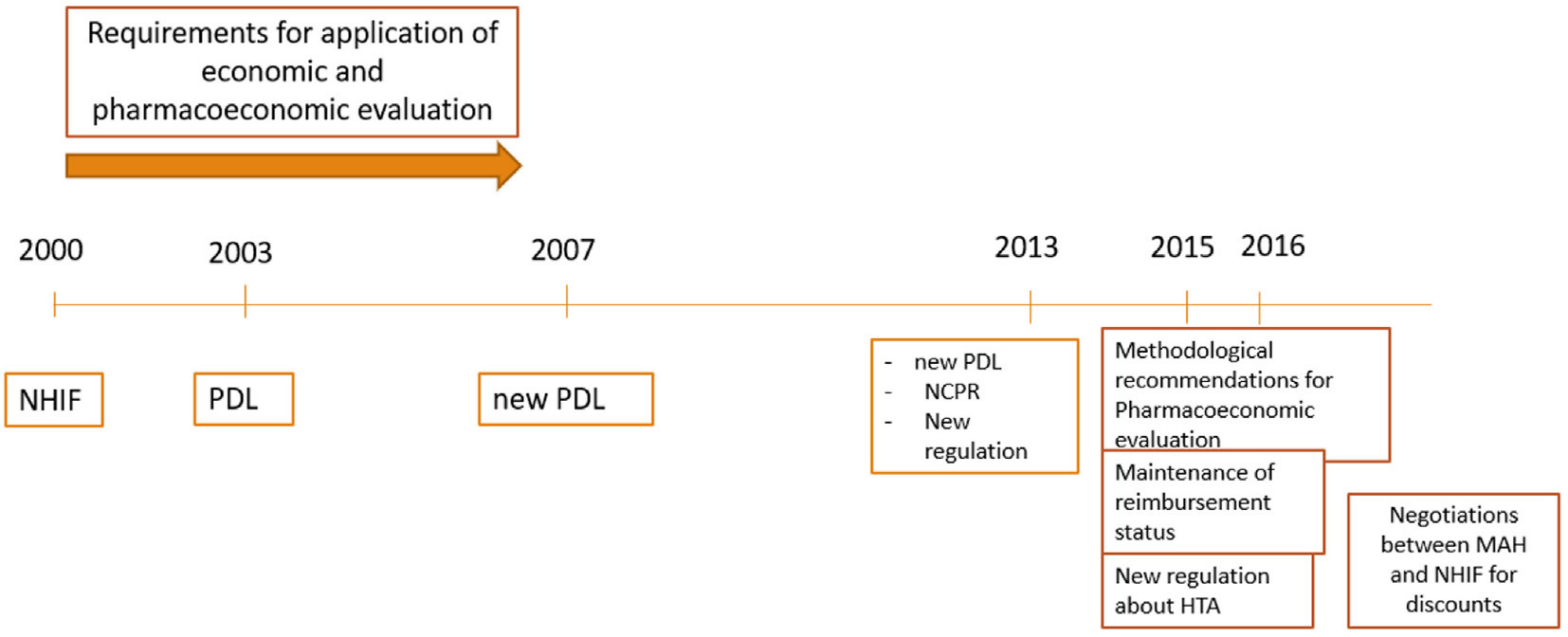
Regulatory development of reimbursement procedures in Bulgaria.

The Health Insurance Act (1998) introduced the mandatory health insurance in Bulgaria (Figure 1) (10). According to this law NHIF was founded in 1999 as an independent public institution (11). The NHIF reimburse MPs, medical devices, dietetic foods, foods for special purposes for treatment of obligatory health insured Bulgarian citizens, as well as for hospitalized patients. For the inclusion of the medicines in the reimbursement lists a methodological approach has been developed and published in 2000, in which several crucial points were stated:

1. economic analysis should precede the pharmacoeconomic analysis;
2. economic analysis includes directs costs, due to product application; market share, prices; additional costs etc.;
3. pharmacoeconomic analysis is a comparison of the costs and consequences of the product application and its competitors (12).

The Council Decree 81 in 2003 stipulates the criteria, conditions and procedures for including MPs in the Bulgarian PDL. Three groups of MPs in PDL were defined:

- new MPs without a medicinal alternative in the clinical practice (new mechanism of action, new ATC code);
- new medicines for which there is a therapeutic alternative with pharmacotherapeutic advantages (group A and B are innovative products);
- MPs with a medicinal alternative in the clinical practice (generics).

A fixed percent of the reimbursement for each MP is defined (100, 75, 50, and 25%) on the basis of its importance for disease therapy and severity of the disease.

In 2007 after the Bulgarian accession to the EU new Regulation was issued and the structure of PDL was changed: ANNEX 1: for fully or partly reimbursed medicines paid by the NHIF; ANNEX 2: medicines paid by the hospital budgets; ANNEX 3: medicines paid by the Ministry of Health budget according to Health Insurance Law; ANNEX 4: medicines for the therapy of rare diseases, HIV, and prophylactics of infections. There were no particular recommendations or guidelines for the development and presentation of the pharmacoeconomic analysis.

The pricing and reimbursement decision process were merged and delegated to one institution in 2013. The National Council on Prices and Reimbursement (NCPR) was established as responsible body for inclusion and exclusion of MPs in the PDL (PDL) and for maintenance of their reimbursement status (13). The PDL was changed and there are now three main annexes and the time for decision was shortened (60 days). All innovative medicines should receive a positive opinion by the Health Technology Assessment Committee since 2015 before issuing the final decision by the Council (14, 15).

**Figure 2:**
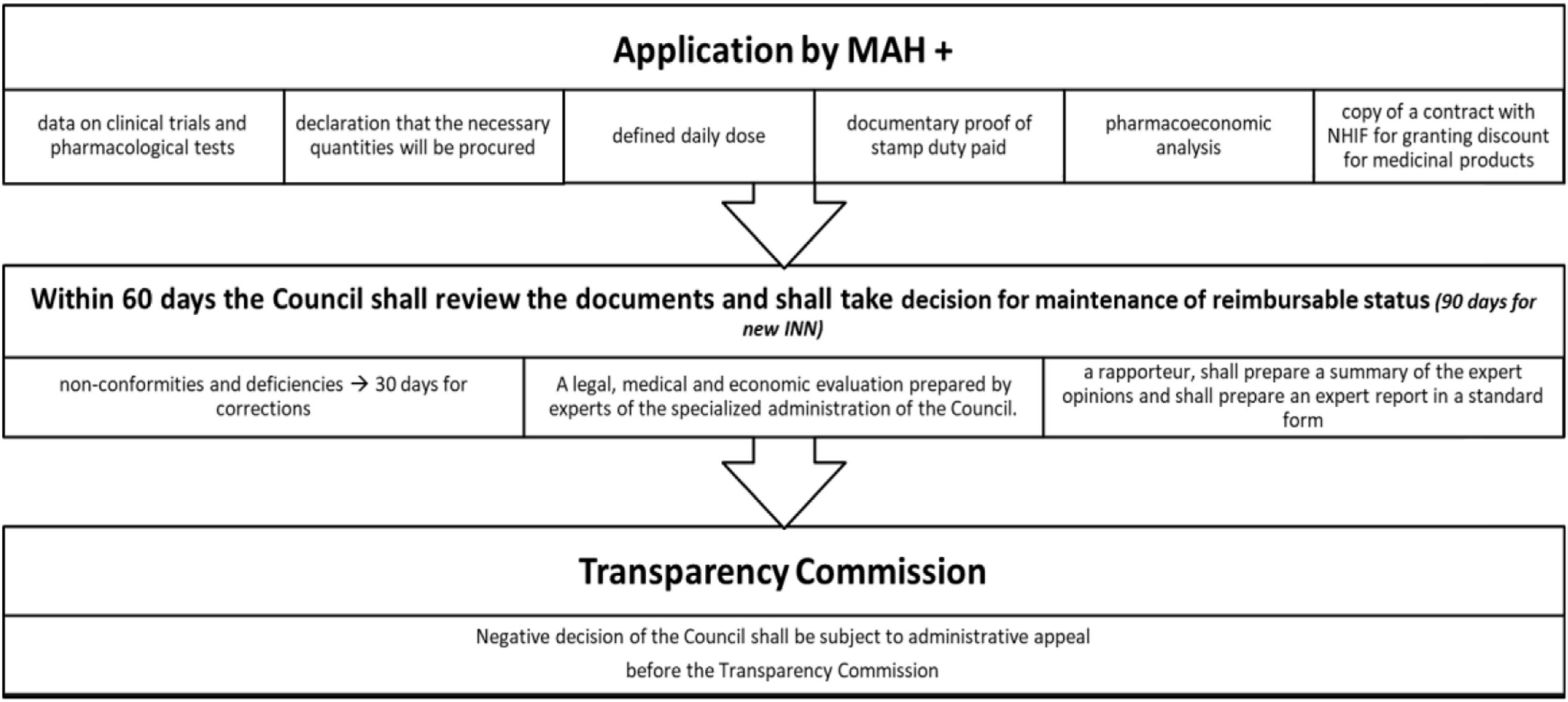
Procedure for inclusion of MPs in PDL. Abbreviations: MPs, medicinal products; PDL, positive drug list.

Pharmacoeconomic and HTA dossiers are prepared following the officially published methodological guidelines. Science-based efficacy, safety, and pharmacoeconomic evidence should be presented in the dossier. Schematic explanation of the reimbursement procedure is shown on Figure 2.

A number of discounts are possible and their level should be negotiated between the Marketing authorization holder (MAH) and the NHIF (16):

1. mandatory discount for reimbursement of Single Source Products (new INNs) (>10%);
2. mandatory discount for new INN and combinations—there is no particular percentage;
3. managed entry agreement—MAH should provide additional discount when the agreed annual expenditures of the MP for each relevant year is exceeded (if the forecast values exceeded to 10% then the discount is not lower than 25%; if the forecast values exceeded to 10–15% the discount is not lower than 50%; if the forecast values exceeded to 15–25 per cent then the discount is not lower than 75%; if the forecast values exceeded 25% then the discount is not lower than 90%);
4. growth discount—MAH should pay back 20% of the relevant rate of growth, when the total growth is higher than 3% from the negotiated (for e.g., the expected expenditures are 100 million BGN, but the real expenditures are 110 mill BGN then the MAH should pay back 20% of 10 million BGN). Exchange rate is 1 BGN = 0.51 Euro;
5. voluntary discounts—for multiple source products; every MAH could provide voluntary additional discounts.

### Access and Affordability to Innovative MPs in Bulgaria

**Figure 3:**
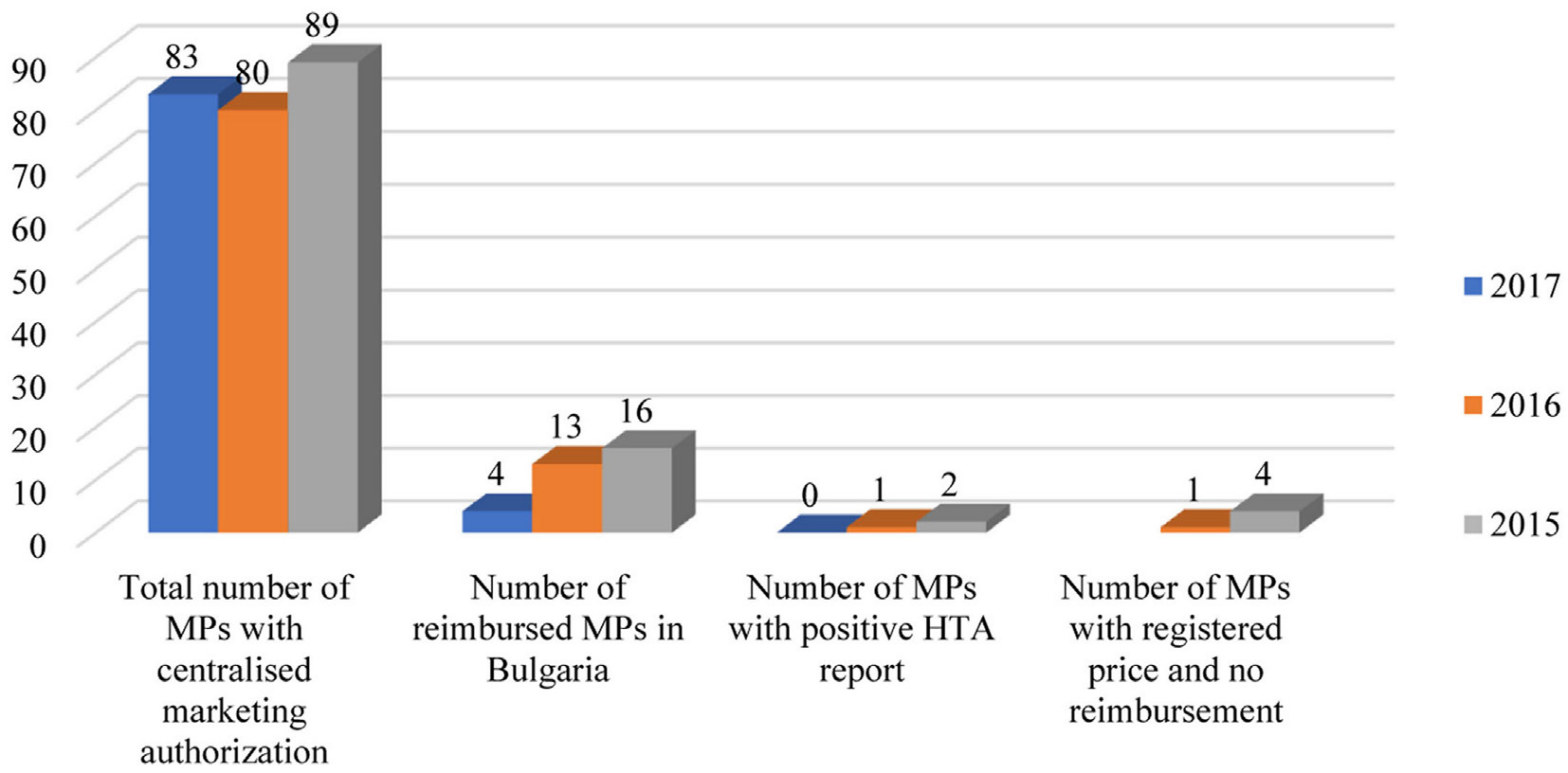
Reimbursement status of MPs in Bulgaria authorized through centralized procedure in the EU. Abbreviations: MA, marketing authorization; MPs, medicinal products; HTA, health technology assessment.

Bulgarian patients have a relatively delayed access to innovative medicines. The percentage of innovative MPs included in the Bulgarian PDL is far below 20%. The number of the newest medicines authorized through the centralized procedure by the EMA in 2017, is 83. Only three of them are reimbursed in Bulgaria and one has received a positive opinion by the HTA Committee. Logically, the number of reimbursed innovative MPs in Bulgaria, which entered the EU-market in 2015 and 2016, is higher than the following year: 18 and 16%, respectively (Figure 3). Some innovative products even do not apply for reimbursement and only register prices for non-reimbursable marketing.

Despite the limited number of reimbursed innovative medicines, very important and promising therapies such as those for Hepatitis C, HIV, multiple myeloma, oncological conditions, etc. are ensured for all Bulgarian patients for whom there is no other option (Table 1).

**Table 1:** Medicinal products with centralized marketing authorization, which are available in Bulgaria.

Active Substance	ATC code	Authorization date	Indication/ICD	Condition Approval/Exceptional Circumstance/Orphan/Generic/Biosimilar	Reimbursement status in Bulgaria, Year
Blinatumomab	L01XC	23/11/2015	ICD C91.0Philadelphia chromosome negative relapsed or refractory B-precursor acute lymphoblastic leukemia (ALL)	Conditional approval; Orphan	Positive HTA; 01.2017
Cobimetinib hemifumarate	L01XE38	20/11/2015	In combination with vemurafenib for the treatment of adult patients with unresectable or metastatic melanoma with a BRAF V600 mutation		Price registration; not reimbursed
Efmoroctocog alfa	B02BD02	19/11/2015	ICD: D66Treatment and prophylaxis of bleeding in patients with hemophilia A		Reimbursed, 2017
Elvitegravir/cobicistat/emtricitabine/tenofovir alafenamide	J05AR	19/11/2015	Treatment of adults and adolescents infected with human immunodeficiency virus 1 (HIV-1) without any known mutations associated with resistance to the integrase inhibitor class, emtricitabine or tenofovir		Price registration; not reimbursed
Sacubitril/valsartan	C09DX04	19/11/2015	ICD: I50.0; I50.1For treatment of symptomatic chronic heart failure with reduced ejection fraction		Reimbursed, 2016
Carfilzomib	L01XX45	19/11/2015	ICD: C90.0Multiple myeloma	Orphan	Reimbursed, 2017
Aripiprazole	N05AX12	16/11/2015	ICD: F20.0, F20.1, F20.5, F20.6, F30.0, F30.1, F31.0, F31.1, F31.2, F31.7Schizophrenia; moderate to severe manic episodes in Bipolar I Disorder; prevention of a new manic episode	Generic	Reimbursed, 2016
Pemetrexed disodium hemipentahydrate	L01BA04	18/09/2015	Malignant pleural mesothelioma	Generic	Reimbursed, 2017
Pregabalin	N03AX16	28/08/2015	ICD: G40.6, G40.7Epilepsy; generalized anxiety disorder	Generic	Reimbursed, 2016
Aripiprazole	N05AX12	20/08/2015	ICD: F20.0, F20.1, F20.5, F20.6, F30.0, F30.1, F31.0, F31.1, F31.2, F31.7Schizophrenia in adults and in adolescents aged 15 years and older.Moderate to severe manic episodes in Bipolar I Disorder and for the prevention of a new manic episode	Generic	Reimbursed, 2016
Bortezomib	L01XX32	20/07/2015	ICD: C90.0, C90.1, C90.2Progressive multiple myeloma	Generic	Reimbursed, 2016
Evolocumab	C10	17/07/2015	ICD: E78.0Hypercholesterolemia and mixed dyslipidaemia		Reimbursed, 2016
Nivolumab	L01XC	19/06/2015	ICD: C43.0, C43.1, C43.2, C43.3, C43.4, C43.5, C43.6, C43.7, C43.8, C43.9Advanced (unresectable or metastatic) melanomaNon-small cell lung cancer (NSCLC)Renal cell carcinoma (RCC)Classical hodgkin lymphoma (cHL)Squamous cell cancer of the head and neck (SCCHN)Urothelial carcinoma		Reimbursed, 2018
Edoxaban tosylate	B01	19/06/2015	ICD: I26.0, I48, I69.3, I69.4, I80.1, I80.2Prevention of stroke and systemic embolism in adult patients with nonvalvular atrial fibrillation (NVAF) with one or more risk factors		Reimbursed, 2017
Empagliflozin/metformin	A10BD20	27/05/2015	ICD: E11.2, E11.3, E11.4, E11.5, E11.9Type 2 diabetes mellitus		Reimbursed, 2016
Netupitant/palonosetron hydrochloride	A04AA	27/05/2015	Prevention of acute and delayed nausea and vomiting		Positive HTA; 08.2017
Ceritinib	L01XE	06/05/2015	Anaplastic lymphoma kinase (ALK) positive advanced non-small cell lung cancer (NSCLC)		price registration; not reimbursed
Bupropion hydrochloride/naltrexone hydrochloride	A08AA	26/03/2015	Management of weight in adult patients (18 years)		price registration; not reimbursed
Secukinumab	L04AC10	15/01/2015	ICD: L40.0, M07.1, M07.2, M07.3, M45.0, M45.1, M45.2, M45.3, M45.4, M45.5, M45.6, M45.7, M45.8Moderate to severe plaque psoriasisPsoriatic arthritisAnkylosing spondylitis		Reimbursed, 2016
Dasabuvir sodium	J05AX16	15/01/2015	ICD: B18.2, K74.0, K74.6Treatment of chronic hepatitis C (CHC) in adultsFor hepatitis C virus (HCV) genotype specific activity		Reimbursed, 2015
Nintedanib	L01XE	15/01/2015	ICD: J84.1Idiopathic pulmonary fibrosis (IPF)		Reimbursed, 2018
Ombitasvir/paritaprevir/ritonavir		15/01/2015	ICD: B18.2, K74.0, K74.6Chronic hepatitis C (CHC) in adultsFor hepatitis C virus (HCV) genotype specific activity		Reimbursed, 2015
Pemetrexed diacid monohydrate	L01BA04	18/01/2016	Malignant pleural mesotheliomaNon-small cell lung cancer		Reimbursed, 2016
Osimertinib mesylate	L01XE	02/02/2016	ICD: C34.0, C34.1, C34.2, C34.3, C34.8, C34.9Locally advanced or metastatic epidermal growth factor receptor (EGFR) T790M mutation-positive non-small-cell lung cancer (NSCLC)		Reimbursed, 2018
Tenofovir disoproxil	J05AF07	08/12/2016	ICD: B18.1, K74.0, K74.6HIV-1 infectionHepatitis B infection	Generic	Reimbursed, 2017
Venetoclax	L01XX52	05/12/2016	ICD: C91.1Chronic lymphocytic leukemia (CLL) in the presence of 17p deletion or TP53 mutation	Conditional approval/orphan	Reimbursed, 2018
Etelcalcetide hydrochloride	H05BX04	11/11/2016	Secondary hyperparathyroidism (SHPT) in adult patients with chronic kidney disease (CKD) on hemodialysis therapy		Reimbursed, 2017
palbociclib	L01XE33	09/11/2016	ICD: C50.0, C50.1, C50.2, C50.3, C50.4, C50.5, C50.6, C50.8, C50.9Hormone receptor (HR) positive, human epidermal growth factor receptor 2 (HER2) negative locally advanced or metastatic breast cancer		Reimbursed, 2018
Tenofovir disoproxil phosphate	J05AF07	15/09/2016	ICD: B18.1, K74.0, K74.6HIV-1 infectionHepatitis B infection	Generic	Reimbursed, 2017
Salmeterol xinafoate/fluticasone propionate	R03AK06	18/08/2016	ICD: J44.8, J45.0, J45.1AsthmaChronic obstructive pulmonary disease (COPD)		Reimbursed, 2017
Elbasvir/grazoprevir	J05A	22/07/2016	ICD: B18.2, K74.0, K74.6Chronic hepatitis C (CHC) in adults		Reimbursed, 2016
Emtricitabine/rilpivirine hydrochloride/tenofovir alafenamide	J05AR19	21/06/2016	Human immunodeficiency virus 1 (HIV-1)		Positive HTA; 08.2017
Sacubitril/valsartan	C09DX04	26/05/2016	ICD: I50.0, I50.1Symptomatic chronic heart failure with reduced ejection fraction		Reimbursed, 2016
Trifluridine/tipiracil hydrochloride	L01BC	25/04/2016	Metastatic colorectal cancer (CRC)		price registration; not reimbursed
Emtricitabine/tenofovir alafenamide	J05AR17	21/04/2016	ICD: B20.0, B20.1, B20.2, B20.3, B20.4, B20.5, B20.6, B20.7, B20.8, B20.9, B21.0, B21.2, B21.3, B21.7, B21.8, B21.9, B22.0, B22.1, B22.2, B22.7, B23.0, B23.1, B23.2, B23.8, B24, Z21Human immunodeficiency virus type 1 (HIV-1)		Reimbursed, 2017
Amlodipine besilate/valsartan	C09DB01	22/03/2016	ICD: I10, I11.0, I11.9, I12.0, I12.9, I13.0, I13.1, I13.2Essential hypertension	Generic	Reimbursed, 2017
Octocog alfa	B02BD02	18/02/2016	ICD: D66Treatment and prophylaxis of bleeding in patients with hemophilia A (congenital factor VIII deficiency)		Reimbursed, 2017
Rituximab	L01XC02	13/07/2017	ICD: C82.0, C82.1, C82.2, C82.7, C82.9, C83.2, C83.3, C83.9, C91.1, M31.3, M31.9Non-Hodgkins lymphoma (NHL)Follicular lymphoma patientsCD20 positive diffuse large B cell non-Hodgkins lymphoma in combination with CHOP (cyclophosphamide, doxorubicin, vincristine, prednisolone) chemotherapyGranulomatosis with polyangiitis and microscopic polyangiitisInduction of remission in adult patients with severe, active granulomatosis with polyangiitis (Wegeners) (GPA) and microscopic polyangiitis (MPA)	Biosimilar	Reimbursed, 2017
Edoxaban tosylate	B01AF03	20/04/2017	ICD: I26.0, I48, I69.3, I69.4, I80.1, I80.2Prevention of stroke and systemic embolismDeep vein thrombosis (DVT) and pulmonary embolism (PE), and prevention of recurrent DVT and PE in adults		Reimbursed, 2017
Tofacitinib citrate	L04AA29	22/03/2017	ICD: M05.0, M05.1, M05.3, M05.8Moderate to severe active rheumatoid arthritis (RA)		Reimbursed, 2018
Darunavir	J05AE10	04/01/2017	Human immunodeficiency virus (HIV-1) infection	Generic	Reimbursed, 2017

*ATC code, Anatomical Therapeutic Chemical Classification System; ICD, International Classification of Diseases*.

### MPs Reimbursement Issues in Bulgaria As an Example for Middle-Income EU Country

The financial limitations of low and middle-income countries are the main drivers for cost-containment measures introduction. In the context of medical and pharmaceutical development, the requirements to the NHIF are increasing. Therefore, more precise and regular financial control mechanisms should be implemented. Another serious problem in these countries is the lack of expertise and the limited local epidemiological data for the purposes of preparing a valuable pharmacoeconomic/HTA dossier. Some of the issues regarding the reimbursement process in Bulgaria and the possible solutions are highlighted in Table 2.

**Table 2:** Reimbursement issues in low and middle-income countries and possible solutions.

Reimbursement process issues	Possible solutions
Financial restrictions (limited budgets)	– Improvement of the collection of health contributions; – Better financial control and monitoring of pharmaceutical expenditures (17); – Improved application of the economic evaluations for the purposes of more efficient reallocation of the resources; – Differentiation of separate budgets for specific group of medicines [for e.g., orphan medicinal products (MPs)].
Lack of expertise (18)	– Providing of educational programs and continuing education for the government employees; – International collaboration.
Improvement in pharmacoeconomic guideline/HTA guideline	– Taking into consideration the latest pharmacoeconomic studies and their implementation into the practice; – Differentiation of the discount levels for both cost and results; – Definition of separate ICER thresholds regarding the type of evaluated MP; – Implementation of multicriteria decision analysis for some specific groups of MPs.
Lack of systems for tracking and assessment of the effectiveness of the MPs	– Dialog between the information technology companies, pharmaceutical industry and health-care policy makers for creation of a unified common information system; – Development and maintenance of patients registries; – Involvement of non-profit patient organization in the HTA process.

## Discussion

The reimbursement policy in Bulgaria could be characterized by implementation of lots of rules for the inclusion of medicines into the PDL and a clear process of reimbursement performed by the National Council on Prices and Reimbursement (19). Despite the necessity of their further improvement, the available pharmacoeconomic and HTA guidelines give the possibility to the policy decision maker to step on a scientific basis in order to make the best possible reimbursement decision. Some problems such as lack of mechanisms for gathering effectiveness data from real-world studies, the periodic legislative changes and the lack of enough experts in the area could be highlighted. Further improvement in the legislative framework is needed in order to cope with the increasing reimbursement expenditures. Collaboration with other European countries could be useful in order to find the best solutions for the reimbursement practice in Bulgaria (20, 21). The process of development and improvement of reimbursement policy is slower, but it could ensure more options for providing innovative medicines to the population (22, 23) as it is the case in other Balkan countries such as Greece (2, 24), Croatia (25), Bosna and Herzegovina, and Republic of Serbia (26). Several crucial changes are proposed in Polish reimbursement system. One of these changes aims to create an innovative reimbursement budget, which will provide funding for reimbursement of innovative products developed by manufacturers with research and development activities with considerable impact on the Polish economy (27). Therefore, the patient access in Poland to innovative therapies could be significantly improved.

Our study confirms that the patient access to innovative medicines from the moment of their marketing authorization is delayed. The number of reimbursed innovative medicines as a percent of the centrally authorized by EMA is far below 20% which confirms some extent of limitations in the patient access. Similar results are presented by Inotai et al. for the patient access to original biologics and biosimilar in Central and Eastern European countries (CEE countries). The authors explain the results with the current implemented biosimilar policies in these countries (28), which means that some improvement in the local legislation is needed. Significant variations exist in uptake of biosimilars in Europe, which could be overcome with implementation of specific procedures and measures (29). While Western Balkan countries has proved through the years that are capable to ensure reimbursed medicines for patients with non-communicable diseases with some exceptions (30), there is still gaps in the knowledge about the patients access to innovative medicines in these countries. Study published in 2017 highlighted the large disparities in access to innovative therapy for metastatic melanoma among the European countries mostly in the Eastern European region (31). The Romanian HTA system implements criteria focused more on the costs and, therefore, it raises a barrier for the innovative medicines in the country (32).

The regulatory bodies especially in CEE countries are pressured in order to ensure new medicines (orphan MPs, innovative biological products, etc.) for severe life-threatening conditions with no available alternative (33). The budget constraints are inevitable, especially in the low- and middle-income countries. The policy makers are trying to balance in the context of deficit resources adopting various approaches. Performance based managed entry agreements for pharmaceuticals is a possible option which is partly applied in Bulgaria. Reassessment of treatments after their inclusion in the reimbursement lists gives a guarantee for collecting of more valuable evidence for effectiveness and cost-effectiveness of the new medicine (34). So, the public fund will be able to stop financing technologies with no proven value in the post reimbursement period. The crucial evidence, which should be taken into account when a reimbursement decision is made, is whether the new medicine brings additional benefits for those patients with no available alternative (23).

Strength of the current study is that it represents the development of Bulgarian reimbursement legislation since its formation in 2000 to these days. This review could be used for the purposes of making more valuable and evidence based decisions for further reforms in the system. As an example of a middle-income Balkan country, the case with Bulgarian reimbursement system could be used as a model for other Balkan countries, which are economically similar to Bulgaria and which are characterized with similar pricing and reimbursement requirements (35). To the best of our knowledge, this is the first study, which makes an attempt to present the access of Bulgarian patients to reimbursed innovative therapies, which received marketing authorization through the centralized procedure in the EU, and to give some recommendations for improvement of the reimbursement decision about these medicines. Further studies could focus more on the real financial burden of the innovative therapies.

## Conclusion

Optimization of the procedure for issuing reimbursement status for innovative MPs is needed especially in the Balkan countries, where lots of issues exist. Improvements in the process of conducting HTA reports and their appraisal, incorporation of adequate systems for following the effectiveness and safety of MPs in the real-world conditions, value-based pricing implementation and increasing the financial control over the health insurance system could be some of the possible solutions. It is crucial the level of expertise in these countries to be enhanced through accreditation of shared master Health Technology Assessment programs. Shared experience among Balkan countries could provide additional valuable information regarding economic evaluation and appropriate reimbursement mechanisms for innovative medicines.

## Author Contributions

All the authors have provided valuable contributions to the manuscript.

## Conflict of Interest Statement

The authors certify that they do not have any conflict of interest to declare regarding the current study.
